# Etiology-Specific Remodeling in Ventricular Tissue of Heart Failure Patients and Its Implications for Computational Modeling of Electrical Conduction

**DOI:** 10.3389/fphys.2021.730933

**Published:** 2021-10-05

**Authors:** Aparna C. Sankarankutty, Joachim Greiner, Jean Bragard, Joseph R. Visker, Thirupura S. Shankar, Christos P. Kyriakopoulos, Stavros G. Drakos, Frank B. Sachse

**Affiliations:** ^1^Nora Eccles Harrison Cardiovascular Research and Training Institute, University of Utah, Salt Lake City, UT, United States; ^2^Department of Biomedical Engineering, University of Utah, Salt Lake City, UT, United States; ^3^Institute for Experimental Cardiovascular Medicine, University Heart Center Freiburg⋅Bad Krozingen, Freiburg, Germany; ^4^Faculty of Medicine, University of Freiburg, Freiburg, Germany; ^5^Department of Physics and Applied Mathematics, School of Sciences, University of Navarra, Pamplona, Spain; ^6^Division of Cardiovascular Medicine, University of Utah School of Medicine, Salt Lake City, UT, United States

**Keywords:** heart failure, cardiac fibrosis, cardiac modeling, electrical conduction, conduction velocity

## Abstract

With an estimated 64.3 million cases worldwide, heart failure (HF) imposes an enormous burden on healthcare systems. Sudden death from arrhythmia is the major cause of mortality in HF patients. Computational modeling of the failing heart provides insights into mechanisms of arrhythmogenesis, risk stratification of patients, and clinical treatment. However, the lack of a clinically informed approach to model cardiac tissues in HF hinders progress in developing patient-specific strategies. Here, we provide a microscopy-based foundation for modeling conduction in HF tissues. We acquired 2D images of left ventricular tissues from HF patients (*n* = 16) and donors (*n* = 5). The composition and heterogeneity of fibrosis were quantified at a sub-micrometer resolution over an area of 1 mm^2^. From the images, we constructed computational bidomain models of tissue electrophysiology. We computed local upstroke velocities of the membrane voltage and anisotropic conduction velocities (CV). The non-myocyte volume fraction was higher in HF than donors (39.68 ± 14.23 vs. 22.09 ± 2.72%, *p* < 0.01), and higher in ischemic (IC) than nonischemic (NIC) cardiomyopathy (47.2 ± 16.18 vs. 32.16 ± 6.55%, *p* < 0.05). The heterogeneity of fibrosis within each subject was highest for IC (27.1 ± 6.03%) and lowest for donors (7.47 ± 1.37%) with NIC (15.69 ± 5.76%) in between. *K*-means clustering of this heterogeneity discriminated IC and NIC with an accuracy of 81.25%. The heterogeneity in CV increased from donor to NIC to IC tissues. CV decreased with increasing fibrosis for longitudinal (*R*^2^ = 0.28, *p* < 0.05) and transverse conduction (*R*^2^ = 0.46, *p* < 0.01). The tilt angle of the CV vectors increased 2.1° for longitudinal and 0.91° for transverse conduction per 1% increase in fibrosis. Our study suggests that conduction fundamentally differs in the two etiologies due to the characteristics of fibrosis. Our study highlights the importance of the etiology-specific modeling of HF tissues and integration of medical history into electrophysiology models for personalized risk stratification and treatment planning.

## Introduction

An estimated 64.3 million people are diagnosed with heart failure (HF) worldwide, incurring an enormous burden on healthcare systems and economies (Bragazzi et al., 2021). The prevalence of HF continues to rise with an aging population in the developed world, and HF incidence is rapidly climbing in developing countries. A broad range of structural or functional cardiac abnormalities is causing or associated with HF. Ischemic heart disease is a major cause, accounting for 26.5% of the HF cases (Bragazzi et al., 2021).

Heart failure and many other heart diseases can be accompanied by fibrosis, which is caused by the increased production of extracellular matrix (ECM) proteins due to the proliferation and differentiation of fibroblasts. ECM deposition as an acute response strengthens the tissue scaffold and replaces cells in the myocardium post-injury. However, chronic fibrotic remodeling adversely affects the ability of the myocardium to propagate electrical signals and circulate blood efficiently.

Cardiac fibrosis is traditionally classified based on the cause that triggered remodeling of the myocardium. Other classifications characterize fibrosis in terms of its location and the nature of remodeling. The condition that led to the remodeling of the myocardium can drive fibrosis to be either reparative or reactive (Silver et al., 1990). Reparative fibrosis, also known as replacement fibrosis, occurs from ischemia and other causes of myocardial injury. Here, the ECM replaces myocytes following necrosis or apoptosis, thereby repairing the scaffold. On the other hand, reactive fibrosis formed in response to various stimuli including pressure overload intersperses with myofibers and is called interstitial fibrosis (González et al., 2018). While reparative fibrosis might result in stable scars, reactive fibrosis can be progressive. Perivascular fibrosis, defined as the increased accumulation of connective tissue around vessels, results from reactive fibrosis and, many times, progresses to interstitial fibrosis (Swynghedauw, 1999).

More recently, fibrosis was characterized by its spatial distribution in the myocardium. A classification of fibrosis in terms of architecture used histological assessment of human cardiac tissue samples, including both ischemic (IC) and nonischemic cardiomyopathies (NIC) (Kawara et al., 2001). Here, fibrosis was categorized as patchy, diffuse, and stringy. Analysis of fibrosis in transmural biopsies from NIC at sub-millimeter scale led to further classification of the architecture as interstitial, diffuse, patchy, and compact (Glashan et al., 2020). Patchy refers to a tightly knit group of fibrotic strands that can be several millimeters long. Diffuse fibrosis consists of less than 1 mm long strands spread over a large area. These strands can form a mesh interspersed with the myocardium or separated with a network of myocardium in between. Stringy fibrosis is composed of thin, long, and well-separated strands homogenously distributed in the tissue. Finally, fibrosis is defined in this classification as compact when the entire transmural section is fibrotic without any viable myocardium.

Fibrosis is commonly quantified by the fractional space occupied by the collagen-specific stain in histology or intensity thresholding in late gadolinium-enhanced magnetic resonance imaging (MRI). The degree of fibrosis estimated by different methods has been clinically correlated with progression, type, and outcome of heart diseases (González et al., 2018; Hinderer and Schenke-Layland, 2019). The increased extracellular volume fraction calculated from cardiac MRI was associated with an increased risk of hospitalization due to HF and death among varied stages of HF and a spectrum of left ventricular ejection fractions (Schelbert et al., 2015). However, a high amount of fibrosis evaluated histologically was associated with death and adverse events only in HF patients with reduced ejection fraction (Aoki et al., 2011). While initially fibrosis was only associated with IC diseases with scars, varied architectures of fibrosis are increasingly associated with NIC diseases (Jellis et al., 2010). Compact fibrosis was found to be rare in NIC, with patchy being the most common. A combination of 2 or 3 types was found in 90% of NIC biopsies (Glashan et al., 2020). Though these observations show the need to assess the heterogeneity of fibrosis for risk stratification, our quantitative understanding of fibrosis and its heterogeneity in human HF is sparse.

65% of HF patients do not survive past 5 years from their initial diagnosis (Bleumink et al., 2021). They are twice as likely to develop arrhythmias compared to the rest of the population, and 50% of mortality is attributed to sudden death primarily resulting from ventricular tachyarrhythmias (Tomaselli and Zipes, 2004; Khurshid et al., 2018). It is well established that fibrosis and structural remodeling in cardiac tissue lead to abnormal conduction patterns (Spach and Boineau, 1997; Nguyen et al., 2014). However, different types of fibrosis can affect conduction differently. For instance, patchy fibrosis contributed to conduction slowing more than stringy and diffuse fibrosis (Kawara et al., 2001).

Computational models of electrical conduction in fibrotic cardiac tissues are extensively used in predicting arrhythmia, especially in determining its origin and features (Trayanova et al., 2018). Personalized models of fibrotic substrates were developed to identify the location for catheter ablation to prevent recurring atrial fibrillations and ventricular tachycardia (Prakosa et al., 2018; Boyle et al., 2019). Commonly, monodomain models of heart tissue are applied, where the myocardium is described as a single continuous domain. Since the extracellular space (ES) is not explicitly described, fibrosis is treated as non-excitable regions with either reduced or no conductivity. Interstitial fibrosis was introduced in such models by decoupling the transverse cellular connections to reflect the distribution of non-conductive collagenous interstitium (Spach et al., 2007). Also, the spatial distribution of fibrosis was modeled by spatially varying the diffusion coefficient of the myocyte domain based on Gaussian random fields (Clayton, 2018). Another model of diffuse fibrosis was introduced as non-conductive collagen randomly distributed in the myocyte layer or myocardial blobs sprinkled in the collagen layer and a fibroblast model coupled with the monodomain model (Chen et al., 2018). Interstitial and patchy fibrosis were modeled based on histological images by only including regions corresponding to myocardium in the simulation of conduction (Campos et al., 2013). In this work, a monodomain model was used and extracellular potentials calculated assuming infinite uniform conductor.

A computationally more demanding alternative to monodomain modeling is bidomain modeling, which comprises a description of the ES (Sepulveda et al., 1989). Here, the ES has an electrical conductivity separate from the myocyte domain. The primary constituent of the ES is conductive interstitial fluid, and ECM proteins contribute only a marginal volume fraction. It is commonly assumed that the interstitial fluid determines the conductivity in the extracellular domain. An expansion of interstitial space is also well documented in different cardiomyopathies (Halliday and Prasad, 2019). In addition to fibrosis and changes in interstitial space, the clefts that separate myocyte sheets modulates the conductivity of ES. The anisotropy of the ES conductivity is well-established and less pronounced than the anisotropy of the myocyte domain (Johnston and Johnston, 2020). Hence the bidomain model is more appropriate to describe features of fibrotic remodeling in cardiac tissues. Fibrosis was modeled as selected nodes in the extracellular domain duplicated and decoupled from the myocyte domain at locations based on collagen in MRI images (Costa et al., 2014). This method was used to simulate interstitial fibrosis (Balaban et al., 2018) and scars (Balaban et al., 2020). Still, image-based microscopic detail of fibrosis is not yet captured in computational models of ventricular tissue in human HF.

We hypothesized that heterogeneity in fibrosis varies in HF patients and the heterogeneity will alter the conduction patterns depending on the etiology of disease. To test this hypothesis, we introduced an approach to quantify the microscopic distribution of fibrosis and its variation within subjects to characterize the heterogeneity of fibrosis. We investigated the relationship between fibrotic remodeling and conduction abnormalities using electrophysiological simulations on image-based meshes of cardiac tissue. For this, we applied confocal microscopic tile scanning of tissue sections from donors and HF patients. Fibrosis was characterized in terms of composition as well as its heterogeneity within each subject. Further, this quantification was used to build electrophysiological models of cardiac tissue to explore the microscopic disturbances in electrical conduction. Finally, we analyzed the influence of different types of fibrosis and the associated HF etiology on electrical conduction velocity (CV) and its dispersion at the microscopic scale.

## Results

### Microstructural Remodeling Distinguishes Heart Failure From Donor Tissues

**FIGURE 1:**
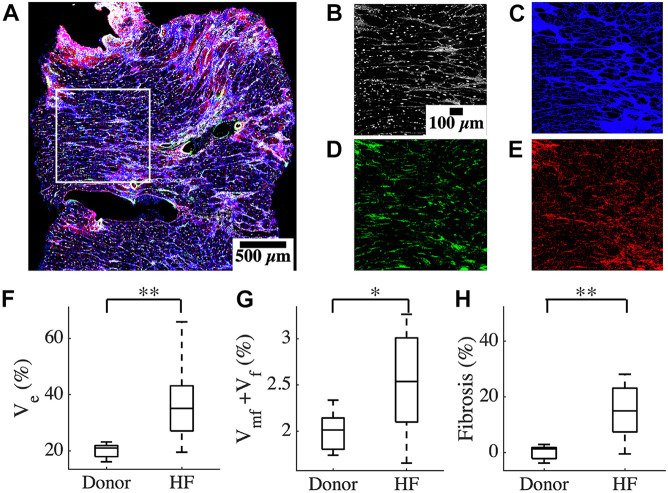
Characterization of human cardiac tissue fibrosis at a microscopic scale. **(A)** Overlay of different fluorescent signals for analysis of fibrosis in an heart failure (HF) tissue sample. The white box marks the 1 mm^2^ region of interest in this sample. The signals are separated and magnified in panels **(B–E)**. **(B)** DAPI signal, **(C)** WGA signal, **(D)** α-SMA, and **(E)** vimentin. **(F)** *V*_*e*_ based on processed WGA signal, and **(G)** *V_*f*_* + *V*_*mf*_ processed from DAPI, α -SMA, and vimentin signals **(H)** Fibrosis in HF. Scale bar in panel **(B)** applies to panels **(C–E)**. *n* = 14 for HF in panel **(G)**. *p < 0.05; **p < 0.01.

We characterized the composition of left ventricular mid-myocardial apical tissue from HF patients (*n* = 16) and donors (*n* = 5) through immunohistochemistry and tile scanning confocal microscopy. Baseline demographic and clinical characteristics of the donor and HF population are presented in Table 1. A tile scan of an HF tissue section with an overlay of the fluorescent signals is illustrated in Figure 1A. The individual signals in a region of 1 mm^2^ area within this overlay are magnified in Figures 1B–E. The nuclei are densely present with varying sizes, reflecting the diversity of cells in cardiac tissue (Figure 1B). The ES labeled by wheat germ agglutinin (WGA) comprises interstitial clefts and larger patches with fibrotic remodeling (Figure 1C). The α-smooth muscle actin (α-SMA) (Figure 1D) and vimentin signals (Figure 1E) correspond to the presence of myofibroblasts (MF) and fibroblasts (F), respectively. We calculated the fractional space occupied by ES (*V*_*e*_), fibroblasts (*V*_*f*_), and myofibroblasts (*V*_*mf*_) by processing the signals. *V*_*e*_ in HF was higher than that of donors (37.49 ± 13.98 vs. 20.1 ± 2.75%, *p* < 5e-4; Figure 1F). The summed *V*_*f*_ and *V*_*mf*_ was higher in HF compared to donors (2.5 ± 0.5 vs. 2.0 ± 0.23%, *p* < 0.05; Figure 1G). Individually *V*_*f*_ and *V*_*mf*_ were not different in HF and donors (Supplementary Figure 1). The total non-myocyte volume fraction (*V*_*nm*_), i.e., the sum of *V*_*e*_, *V*_*f*_, and *V*_*mf*_, was higher in HF than donors (39.68 ± 14.23 vs. 22.09 ± 2.72%, *p* < 5e-4). The fibrosis in HF, calculated as the difference between *V*_*nm*_ in HF and the mean *V*_*nm*_ of donors, was 17.59 ± 14.23% (Figure 1H).

**TABLE 1:** Baseline demographic and clinical characteristics of donor and heart failure (HF) population.

	**Donor (*n* = 5)**	**HF (*n* = 16)**		
		**All HF**	**IC (*n* = 8)**	**NIC (*n* = 8)**
Age (years)	51.2 ± 13.48	53.44 ± 10.28	58.50 ± 6.74^$^	48.38 ± 11.07
Male, *n* (%)	2 (40)	13 (81.25)	7 (87.50)	6 (75)
Height (cm)	161.03 ± 8.90	176.67 ± 8.27	178.77 ± 5.95	174.56 ± 10.06
Weight (kg)	68.12 ± 13.23	93.55 ± 22.8	101.82 ± 19.53	85.28 ± 24
BMI (kg/m^2^)	26.01 ± 2.91	29.94 ± 7.11	31.98 ± 6.33	27.89 ± 7.67
LVEF (%)	71.40 ± 7.02	21.56 ± 11.25	24.38 ± 10.5	18.75 ± 11.96
NYHA class III, *n* (%)		5 (31.25)	3 (37.50)	2 (25)
NYHA class IV, *n* (%)		11 (68.75)*	5 (62.50)	6 (75)
Duration of HF (months)		62.81 ± 53.04	61.75 ± 53.69	63.88 ± 56.05
LVAD, *n* (%)		11 (68.75)	5 (62.50)	6 (75)
Direct transplant, *n* (%)		5 (31.25)	3 (37.50)	2 (25)

*Values are shown in *n* (%) or mean ± SD when appropriate.*

*^$^*p* < 0.05 between IC and NIC.*

*NYHA class for an IC patient was III or IV and is considered IV here.

*BMI, body mass index; LVEF, left ventricular ejection fraction; NYHA, New York Heart Association.*

### Heterogeneity of Fibrosis Differentiates Heart Failure Etiologies

**FIGURE 2:**
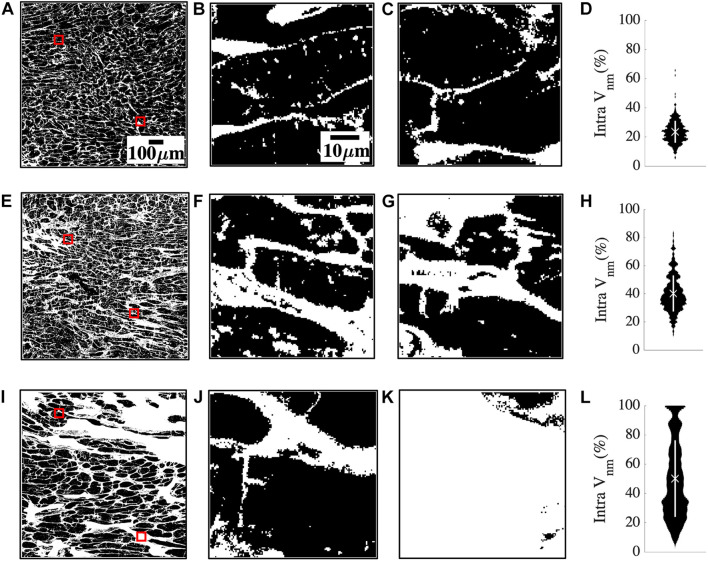
Quantification of heterogeneity of *V*_*nm*_ in a representative donor **(A–D)**, mean fibrosis HF **(E–H)**, and high fibrosis HF **(I–L).** **(A)** 1 mm^2^ region of segmented non-myocyte space in white in a donor tissue section with two of the 400 sub-images of size 50 × 50 μm^2^ highlighted with red boxes. **(B,C)** Zoom-ins of regions within red boxes in panel **(A)** revealed normal myocyte membranes and capillaries. **(D)** The probability distribution of *V*_*nm*_ in all the sub-images of panel **(A)** was narrowly spread. The mean marked with “X” aligned with the distribution peak, and SD marked with the white line segment was small. **(E)** Region of interest in HF tissue with fibrosis similar to the mean of all HF. **(F,G)** Magnified sub-images within red boxes in panel **(E)** indicated enlarged ES. **(H)** Though the probability distribution was spread wider than panel **(D)**, the mean *V*_*nm*_ was close to the peak. **(I)** Region of interest in a high fibrosis HF tissue. **(J,K)** Magnified sub-images within red boxes in panel **(I)** were very different from each other. **(J)** Had enlarged interstitial ES while panel **(K)** was highly fibrotic and devoid of any myocytes. **(L)** The distribution was more uniformly spread out than panel **(H)** with the peaks away from the mean. Scale bar in panel **(A)** applies to panels **(E,I)**. Scale bar in panel **(B)** applies to panels **(C,F,G,J,K)**.

The large spread of fibrosis in HF motivated further investigation of variability in intra-subject non-myocyte fractions (*intra V_*nm*_*). We calculated *V*_*nm*_ of each 50 × 50 μm^2^ sub-image within the overall region of analysis in each subject and obtained their probability distribution. Subregions within a donor sample were similar to each other (Figures 2A–C). The two representative sub-images indicate normal myocyte dimensions (Figures 2B,C). *Intra V_*nm*_* was 23.54 ± 7.54% with its distribution concentrated near the mean (Figure 2D). A subject with NIC whose *intra V_*nm*_* of 40.25 ± 12.85% is close to the mean *V*_*nm*_ = 39.68% of all HF revealed sub-images with enlarged ES (Figures 2E–G). The interstitial space varied but was expanded and distinct from that of the donor. Though the *V*_*nm*_ distribution spread wider than the donor, its peak was near the mean (Figure 2H). Images from an HF subject with IC etiology and an *intra V_nm_* = 50.01 ± 34.61% revealed patches of fibrosis covering the entire area in many sub-images that were devoid of any myocytes (Figures 2I–L). While some sub-images displayed the myocytes and ES similar to the donor sample and some others reflected the average fibrosis with thickened collagen deposits and hypertrophic myocytes, many sub-images with 100% *V*_*nm*_ shifted the distribution. The distribution covered almost the entire range, peaking between 20 and 40% and again, at 100%, all distinct from the mean *V*_*nm*_ (Figure 2L).

### Characteristics of Fibrosis Distinguished Between Ischemic and Nonischemic Etiologies

**FIGURE 3:**
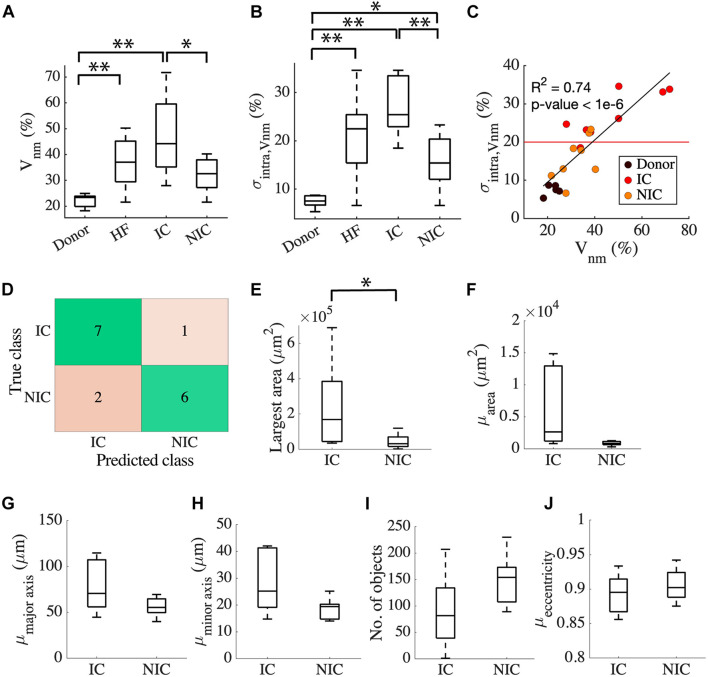
Classification of HF by fibrosis characteristics relates to disease etiology. **(A)** *V*_*nm*_ of the donor has an interquartile range (IQR) of ∼5% compared to HF with an IQR of ∼30%. HF split to IC (*n* = 8), and NIC (*n* = 8) revealed an IQR of ∼10% for NIC and ∼35% for IC. **(B)** σ*_*intra,Vnm*_* has a median <10% for donors, >20% for IC, and ∼15% for NIC. **(C)** Strong relationship between σ*_*intra,V*__*nm*_* vs. *V*_*nm*_ displayed by the linear regression represented by the black line segment. The red line segment shows that σ*_*intra,V*__*nm*_* = 20.58% discriminated most IC from NIC. **(D)** Classification of HF by *k*-means algorithm applied to σ*_*intra,V*__*nm*_* was 81.25% accurate. Connected component analysis of fibrotic objects in images showed that **(E)** area of the largest object was larger in IC than NIC. The **(F)** mean area and the mean lengths of **(G)** major and **(H)** minor axes of the patches were not different for IC and NIC. IC and NIC were not differentiated by **(I)** the number of isolated fibrotic objects with area > 150 μm^2^ or **(J)** the eccentricity of the patches. *p < 0.05; **p < 0.01. Outliers in HF are not indicated in panel **(A)**.

The clinical characterization of two distinct groups of HF prompted us to investigate the relationship between the etiology of the disease and the characteristics of fibrosis based on *intra V_*nm*_*. Splitting HF into IC (*n* = 8) and NIC (*n* = 8) revealed that the large spread of *V*_*nm*_ of HF is primarily due to the IC (Figure 3A). *V*_*nm*_ was higher for IC vs. NIC (47.2 ± 16.18 vs. 32.16 ± 6.55%, *p* < 0.05). However, it did not differ between NIC and donor groups. We further analyzed the standard deviation (SD) of *intra V_*nm*_* (σ*_*intra,Vnm*_*) between these groups (Figure 3B). As expected, σ*_*intra,Vnm*_* was higher in HF vs. donor (21.4 ± 8.2 vs. 7.47 ± 1.37%, *p* < 5e-6). Additionally, this measure was not only different between IC and NIC (27.1 ± 6.03 vs. 15.69 ± 5.76%, *p* < 0.05), but also between NIC and donor (15.69 ± 5.76 vs. 7.47 ± 1.37%, *p* < 0.05). Regression analysis revealed a strong relationship between σ*_*intra,Vnm*_* and *V*_*nm*_ (*R*^2^ = 0.74, *p* < 1e-6; Figure 3C). Separately, IC samples corresponded to a 3% increase in σ_*intra,Vnm*_ for every 10% increase in *V*_*nm*_. Comparatively, the increase in σ*_*intra,Vnm*_* for the NIC samples was 5.4% for every 10% increase in *V*_*nm*_. The donors, in contrast, formed a cluster with low *V*_*nm*_ and low σ*_*intra,Vnm*_*. Overlap of IC and NIC samples was present in a region for *V*_*nm*_ between 30 and 40% and σ*_*intra,Vnm*_* between 15 and 25%.

Since σ*_*intra,Vnm*_* was markedly different between IC and NIC, we used it as a feature to cluster HF using a *k*-means algorithm. The centroids of clusters calculated from the *k*-means algorithm was used to assign the true labels. 7 out of 8 IC and 6 out of 8 NIC were correctly classified (Figure 3D). A σ*_*intra,Vnm*_* of 20.58%, which is the mean of the centroids of the two clusters separated IC and NIC (Figure 3C). Further, we explored the geometrical features of the fibrotic patches extracted from the binary image of *V*_*nm*_ for each subject that can distinguish between IC and NIC. By analyzing the properties of connected components of the images that have areas larger than 150 μm^2^, we assessed the area of the largest component (Figure 3E) and the mean area of all the components in an image (Figure 3F). By fitting the individual components to ellipses, we also calculated the mean length of the major axis (Figure 3G), minor axis (Figure 3H), and mean eccentricity of the components (Figure 3I). The connected components in each image were counted as well (Figure 3J). The largest fibrotic patch area was higher in IC than NIC (23.96e4 ± 24.46e4 vs. 4.39e4 ± 4.09e4 μm^2^, *p* < 0.05). The other features were not different between both groups. Both, IC and NIC images exhibited many distinct elongated fibrotic patches.

We compared the ability of σ*_*intra,Vnm*_* to discriminate between IC and NIC samples with that of *V*_*nm*_ and the area of the largest fibrotic patch. The comparison was based on accuracy, sensitivity, specificity, positive, and negative predictive value (Table 2). The accuracy was highest (81.25%) when σ*_*intra,Vnm*_* alone was used as the clustering feature. While the specificity and positive predictive value was 100% for the other two classifiers, the low values for sensitivity and negative predictive values make them less effective. Applying the algorithm on a combination of these features did not yield higher accuracy than σ*_*intra,Vnm*_* alone.

**TABLE 2:** Comparison of the binary clustering of HF as ischemic (IC) and nonischemic (NIC) cardiomyopathy by *k*-means algorithm applied to different features from image analysis.

**Clustering features**	**Accuracy (%)**	**Sensitivity (%)**	**Specificity (%)**	**Positive predictive value**	**Negative predictive value**
σ*_*intra,Vnm*_*	81.25	87.5	75	0.78	0.86
*V*_*nm*_	75	50	100	1.00	0.67
Area of largest fibrotic patch	62.5	25	100	1.00	0.57

### Patterns of Conduction Have Pronounced Variation in Different Heart Failure Etiologies

**FIGURE 4:**
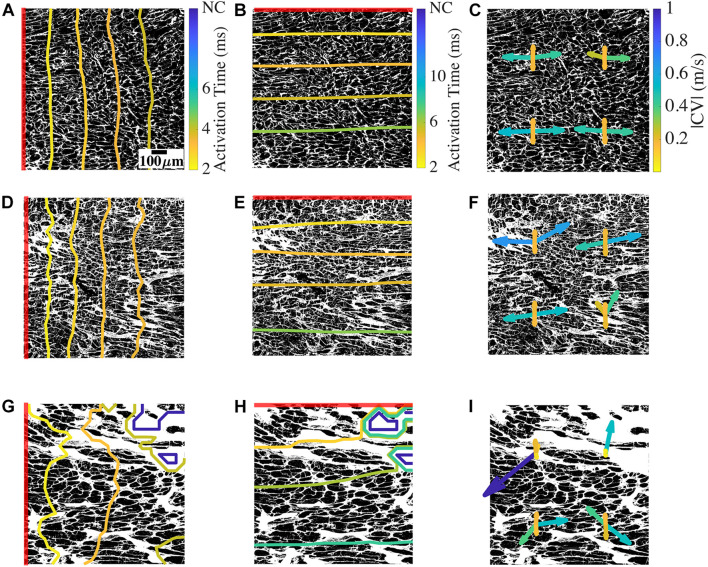
Conduction and CV in bidomain model based on a representative donor, low fibrosis HF, and high fibrosis HF tissue. **(A)** Longitudinal conduction in an example donor tissue shown by activation times as contour plot overlayed on the non-myocyte fraction from which the mesh was generated. **(B)** Transverse conduction in the donor. **(C)** CV vectors at four positions in the mesh. At each position, two CV vectors correspond to longitudinal conduction from left to right and right to left, and two CV vectors correspond to transverse conduction from top to bottom and bottom to top. **(D–F)** Conduction and CV vectors for low fibrosis HF. **(G–I)** Longitudinal and transverse conduction and CV vectors for high fibrosis HF. The red line segment in each image shows the location of the applied stimulus. NC indicates no conduction. Scale bar in panel **(A)** applies to all figures. Color bar in panel **(A)** applies to panels **(D,G), (B)** applies to panels **(E,H)**, and **(C)** applies to panels **(F,I)**.

We evaluated the characteristics of action potential conduction using a bidomain model based on 2D images of donor and HF tissues. By applying a line stimulus based on voltage clamping for 2 ms, the local activation and CV were assessed over the domain. Example simulations in Figure 4 revealed the relationship between the conduction and the underlying microstructure. We overlaid the activation time contours on representative segmented binary WGA images. We also overlaid CV vectors on the WGA images at the locations where they were calculated in the mesh. The longitudinal and transverse conduction was evaluated separately by placing the stimulus corresponding to the myocyte orientation in the images.

For the mesh generated from a donor tissue sample image, a line stimulus at the left edge of the domain initiated longitudinal conduction aligned with the orientation of the long axis of myocytes (Figure 4A). The activation wavefronts through the domain at different timepoints were primarily smooth and parallel to the stimulus. The complete domain was activated at ∼4.4 ms (Supplementary Movie 1). A line stimulus at the top edge of the same mesh generated transverse conduction (Figure 4B). The transverse wavefronts were again smooth and parallel to the stimulus, and the activation was completed at ∼7.4 ms. The simulation of longitudinal conduction was also performed by placing the stimulus at the right edge of the domain. Similarly, transverse conduction was repeated by placing the stimulus at the bottom edge.

We evaluated the CV vectors in the four simulations for each mesh at four uniformly distributed points by calculating its magnitude (|CV|) and tilt (∠CV) from the direction normal to the line stimulus. An example is shown in Figure 4C. The four CV vectors at each location correspond to the four directions of conduction, i.e., two longitudinal CVs (CVLs) and two transverse CVs (CVTs). The four CV vectors in the donor mesh were similar to each other, except for a vector for the right to left longitudinal conduction. The magnitudes of CVL (|CVL|) ranged between 0.26 and 0.54 m/s, and the tilt angles of CVL (∠CVL) varied from −15° to 2°. |CVT| were 0.16–0.18 m/s and ∠CVT ranged from −0.2° to 2.9°. The direction of vectors corresponding to the conduction from left to right and right to left was mostly, but not always, mirrored.

In simulations with an example mesh from NIC tissue, the wavefronts of longitudinal conduction were distinct from the donor because of their complex geometry (Figure 4D). The wavefront lost its smoothness, especially in regions with prominent fibrotic patches. The conduction was faster than the donor, with the activation completed at 3.9 ms (Supplementary Movie 2). The transverse conduction, in this case, completed activation slightly faster than the donor sample at 7 ms (Figure 4E). The wavefronts were mainly parallel to the stimulus. In this HF sample with low fibrosis and restricted chiefly to interstitial fibrosis, |CVL| from 0.23 to 0.68 m/s spanned a more extensive range than that of the donor (Figure 4F). ∠CVL of −61.2° to 11° presented remarkably more tilt than that of the donor, and the differences between conduction in opposite directions were more pronounced. The bottom right location surrounded by fibrotic patches illustrates this variation in CVL (Figure 4G). |CVT| of 0.16–0.2 m/s and ∠CVT of −0.38° to 2.3° in this example of NIC was similar to that of the donor.

In high fibrosis HF with IC etiology, the longitudinal wavefronts were heavily distorted with conduction blocked at the top right corner. The conduction was delayed, with activation completed for the rest of the domain at 4.7 ms. The wavefront for transverse conduction was heavily distorted in the beginning due to the block. The wavefront regained the smoothness and became parallel to the stimulus as conduction reached near the end of the mesh (Figure 4H). The activation was completed for conductive regions of the domain at 8.5 ms, slower than the donor and NIC samples (Supplementary Movie 3). |CVL| of 0.01–1.11 m/s in this example of IC displayed even larger variations in longitudinal conduction than NIC (Figure 4I). ∠CVL of -90.29° to 89.59° illustrated some CV almost perpendicular to the original direction of conduction. The difference in the direction of CV vectors between the opposing conduction was particularly evident. The CVL at the top left corresponding to conduction beginning at the left end was very small and almost parallel to the stimulus. In contrast, the local instantaneous CVL for the opposite conduction was larger than 1 m/s but less deviated. Similarly, the two CVL vectors of opposing direction of conduction at the top right differed since they were at the edge of the conductive region. Though both their directions are 90° from the expected conduction direction, one had a magnitude ∼0.5 m/s, and the other was close to 0 m/s. The two lower sets of CV vectors are comparable to those observed in Figure 4F. |CVT| of 0.04–0.52 m/s and ∠CVT of −3.26° to 167.99° indicated magnitudes at the top two locations much smaller than the bottom two locations as well as the donor and NIC cases. The vector for conduction from left to right near the block was large and opposite to the conduction direction.

By analyzing activation times in all the samples, we found that 9 out of the 16 HF models had at least one non-conductive region. In two HF models, longitudinal conduction in either direction was fully blocked and did not reach the opposite end. In addition, transverse conduction in one direction of an HF mesh was blocked before reaching the opposite end.

To evaluate boundary effects on our simulation and calculation of CV, we expanded the computational domain for a donor tissue sample (Supplementary Figure 4A). The domain was extended by incorporating meshes with homogenous volume fractions to the left and right of the donor mesh (Supplementary Figure 4B). Similar as described above, we performed simulations by applying the voltage clamp on the left and right end of this domain to calculate longitudinal conductivities at the same four locations as the original donor mesh. However, because of the increased length of the domain, the measurement of CV was more than 2 mm away from the boundary. We obtained a mean error of 0.00125 m/s for |CVL| and −1.5° for ∠CVL between the original and extended meshes. The root mean square error was 2.04 and 2.79% for |CVL| and ∠CVL, respectively. This example indicates that boundary effects are marginal.

### Etiology-Dependent Heterogeneity of Conduction Velocity Vectors

**FIGURE 5:**
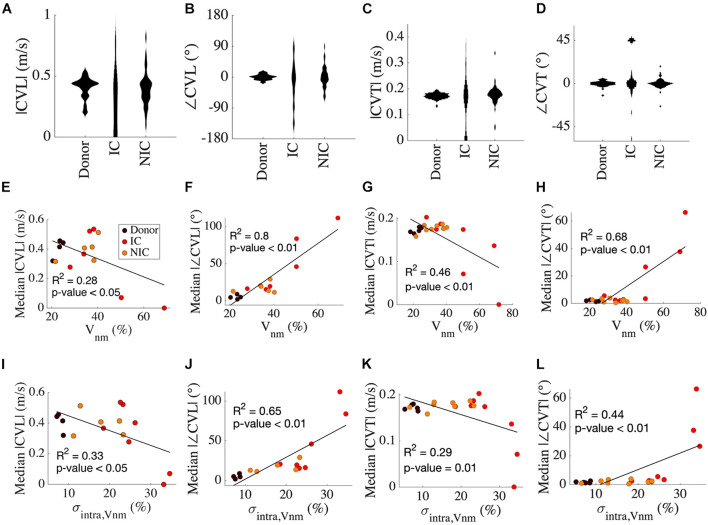
Analysis of CV vectors in donors, IC, and NIC. **(A)** The probability distribution of |CVL| was widely spread in IC compared to NIC and donors. In donors, |CVL| clustered at ∼0.4 m/s in. Three outliers for IC between 1 and 3 m/s are not shown. **(B)** ∠CVT is more widely spread in IC than NIC and is marginal in donors. **(C)** |CVT| peaks just below 0.2 m/s for donors and NIC, with NIC having a greater spread and IC spread out similar to CVT. Three outliers in IC greater than 0.5 m/s are excluded. **(D)** ∠CVT are much smaller than ∠CVL but exhibit a similar spread in the three groups. **(E)** Median |CVT| for each subject decreased with increasing ∠CVT. **(F)** The median |∠CVL| increased with *V_*n*__*m*_.* **(G)** The median |CVT| and **(H)** median |∠CVT| did not vary for *V*_*nm*_ < 40% and were similar to that of CVL. **(I–L)** Similar relationships of CV with σ*_*intra,V*__*nm*_* as from *V*_*nm*_ in panels **(E–H)**. Legend in panel **(E)** applies to panels **(F–L)**.

We calculated the four CV vectors at four locations from all the HF and donor samples to evaluate the distribution of CV vectors and their relationship with fibrosis. While the mean |CVL| of donors, IC, and NIC were 0.4 m/s, the probability distribution of CV vectors exhibited a large spread in HF vs. donors (Figure 5A). |CVL| was more widely spread in IC compared to NIC (0.4 ± 0.49 vs. 0.4 ± 0.14 m/s) whereas it was more compact in donors than NIC (0.4 ± 0.09 vs. 0.4 ± 0.14 m/s). Donors had a distinct peak in the distribution that is close to its mean. SD in |CVL| was three times larger in IC than NIC and less than NIC in donors. |CVL| for conduction from left to right and right to left for donors was similar, but less symmetric for NIC (Supplementary Figure 2A).

Tilt angles of CVL was most pronounced for IC, much smaller for NIC, and negligible in donors (−16.85° ± 62.85° vs. 0.66° ± 32.03° vs. 0.03° ± 7.94°) (Figure 5B). In IC, the direction of the CV vector reversed with respect to the conduction in a few cases. SD in ∠CVL of IC was nearly twice that of NIC, and that in donors was a quarter of NIC. The symmetry of conduction in the two opposite directions in donor was also reflected in low ∠CVL (Supplementary Figure 2B). This symmetry was lost in IC and NIC. The heterogeneity in CVL is corroborated by the spread of maximal upstroke velocities in longitudinal conduction for the three groups (Supplementary Figure 3A). While maximal upstroke velocity in donors clustered at ∼200 V/s, the maximal upstroke velocity in IC and NIC was more widely spread. The absence of activation was present only in IC.

|CVT| was marginally larger in IC than NIC (0.23 ± 0.42 vs. 0.18 ± 0.07 m/s) and the mean coincided with the peak of their distribution for NIC and donors (Figure 5C). The mean |CVT| for donors at 0.17 ± 0.01 m/s was similar to NIC. The wide spread of |CVT| in IC and the distinct lump at 0 m/s, indicating no conduction, was similar to that of |CVL|. The SD of |CVT| was seven times larger for IC vs. NIC and six times larger for NIC vs. donors. The symmetry between the two directions of transverse conduction was similar to longitudinal conduction in donors. The |CVT| symmetries were higher for IC and NIC than |CVL| symmetries (Supplementary Figure 2C). ∠CVT was much smaller than ∠CVL for all groups, with the tilt angles limited between −45° to +45° (Figure 5D). ∠CVT was most prominent for IC, smallest for NIC, and negligible for donors (−12.37° ± 53.76° vs. –0.09° ± 5.04° vs. 0.22° ± 3°). The spread was smallest in donors, largest in IC, and in the middle for NIC. The SD of ∠CVT in IC was ten times larger than in NIC. The asymmetry in ∠CVT was pronounced in IC (Supplementary Figure 2D). The maximal upstroke velocity in transverse conduction peaked at ∼250 V/s for both donor and NIC groups, though NIC had a greater spread of values similar to longitudinal conduction (Supplementary Figure 3B). We performed f-tests between donors, IC and NIC groups for |CVL|, |CVT|, ∠CVL, and ∠CVT and found that variances were different in all comparisons (*p* < 0.01).

Results of linear regression analysis between variables of CV and our fibrosis measures are summarized in Table 3. Median |CVL| for each subject decreased with increasing *V*_*nm*_ of the subjects (Figure 5E). Median absolute value of ∠CVL (|∠CVL|) exhibited a strong positive linear relationship with *V*_*nm*_ (Figure 5F). Median |CVT| decreased (Figure 5G) and median absolute value of ∠CVT (|∠CVT|) increased (Figure 5H) as *V*_*nm*_ increased. |∠CVL| increased by 2.1° and |∠CVT| increased by 0.91° per 1% increase in fibrosis. While the median |CVL| and |CVT| displayed negative relationships with σ*_*intra,Vnm*_* similar to that with *V*_*nm*_ (Figures 5I,K), median |∠CVL| and |∠CVT| displayed weaker relationship with σ*_*intra,Vnm*_* than *V*_*nm*_ (Figures 5J,L).

**TABLE 3:** Results of linear regression analysis between conduction velocities (CV) and fibrosis.

***x* variable**	***y* variable**	**Model for linear regression**	**Coefficient of determination (*R*^2^)**	**Significance vs. constant model (*p*)**
*V*_*non–myo*_	Median |CVL|	*y* = -0.01*x* + 0.58	0.28	0.03
*V*_*non–myo*_	Median |∠CVL|	*y* = 2.10*x* - 48.69	0.80	3.1e-6
*V*_*non–myo*_	Median |CVT|	*y* = -0.002*x* + 0.24	0.46	7.3e-4
*V*_*non–myo*_	Median |∠CVT|	*y* = 0.91*x* - 24.27	0.68	4.8e-6
σ*_*intra–subject,Vnon–myo*_*	Median |CVL|	*y* = -0.01*x* + 0.54	0.33	0.02
σ*_*intra–subject,Vnon–myo*_*	Median |∠CVL|	*y* = 2.75*x* – 25.84	0.65	1.7e-4
σ*_*intra–subject,Vnon–myo*_*	Median |CVT|	*y* = -0.003*x* + 0.21	0.29	0.01
σ*_*intra–subject,Vnon–myo*_*	Median |∠CVT|	*y* = 1.15*x* - 12.69	0.44	9.6e-4
σ*_*intra–subject,Vnon–myo*_*	σ_|__∠CVL|_	*y* = 1.40*x* – 4.49	0.78	6.6e-6
σ*_*intra–subject,Vnon–myo*_*	σ_|__∠CVT|_	*y* = 1.79*x* - 18.77	0.59	4.3e-5
σ*_*intra–subject,Vnon–myo*_*	σ_|CVL|_	*y* = 0.01*x* + 0.001	0.18	0.1
σ*_*intra–subject,Vnon–myo*_*	σ_|CVT|_	*y* = 0.01*x* – 0.04	0.11	0.13
*V*_*non–myo*_	|CVL| /|CVT|	*y* = 0.01*x* + 1.89	0.07	0.35
σ*_*intra–subject,Vnon–myo*_*	|CVL| /|CVT|	*y* = -0.01*x* + 2.41	0.06	0.41

σ_|CVL|_ and σ_|*CVT*|_ did not have any relationship with σ*_*intra,Vnm*_* (Supplementary Figures 3C,D). In contrast, σ_|__∠CVL|_ and σ_|__∠*CVT*|_ displayed strong positive relationship with σ*_*intra,Vnm*_* (Supplementary Figures 3E,F). The anisotropy |CVL| / |CVT| in all the cases and the three groups ranged between 1 and 3. The anisotropies were not affected by *V*_*nm*_ or σ*_*intra,Vnm*_* (Supplementary Figures 3G,H).

## Discussion

This study revealed fundamental differences in microscopic electrical conduction in ventricular tissues in normal human hearts and hearts from end-stage HF patients of different etiologies. These differences in conduction were caused by fibrotic remodeling that varied with HF etiology. The heterogeneity of fibrosis in HF discriminated the etiology with a high accuracy of 81.25%. We quantified fibrosis considering the cellular constituents, i.e., fibroblasts and myofibroblasts, in addition to the ES, and also analyzed intra-subject heterogeneity of fibrosis at a microscopic scale. Commonly, this microscopic heterogeneity is not assessed in fibrosis quantifications, but it strongly affected outcomes of computational modeling of conduction in HF tissues.

### Heterogeneity of Fibrosis in Heart Failure

Current histological methods for quantifying myocardial fibrosis rely exclusively on the collagen content, and clinical imaging methods cannot provide information on fibrosis at the microscopic scale. Most measures of fibrosis used to parameterize conduction models do not account for fibroblasts and myofibroblasts that are major cellular constituents of the remodeling. Our past work on the analysis of fibrosis in healed myocardial infarction in an animal model showed a significant increase in fibroblasts and myofibroblasts in the scar border zone (Greiner et al., 2018). While the *V_*f*_* + *V*_*mf*_ was 9% proximal to the scar, it dropped to ∼5% at a distance ∼1 mm from the scar. Here, our analysis indicates that *V*_*f*_ + *V*_*mf*_ increased in the end-stage HF vs. donors, but to a smaller extent than in the infarction model. A potential explanation is that the tissue is not undergoing active remodeling since most samples are from chronic HF patients (Table 1).

The increase in σ*_*intra,Vnm*_* was related to the increase in *V*_*nm*_ across all samples. Separately the samples of NIC showed a stronger effect of *V*_*nm*_ on σ*_*intra,Vnm*_*, while the samples of IC revealed smaller increases in σ*_*intra,Vnm*_* with increase in *V*_*nm*_. Hence, the increase in heterogeneity not only corresponded to the amount of fibrosis but also the HF etiology. A study on fibrosis in tetralogy of Fallot showed that intra-subject variability was similar to the inter-subject variability in single 2D histological thereby limiting the quantification of fibrosis in a truly representative manner (Wuelfers et al., 2021). In our results, the intra-subject variability was higher than inter subject variability of all HF. The recovery of cardiac function in HF patients caused by mechanical unloading using left ventricular assist devices (LVADs) has been previously associated with an increase in fibrosis (Drakos et al., 2010). However, specific changes in composition and distribution of fibrosis underlying this change remain unknown. We suggest that quantifying the heterogeneity of fibrosis will increase understanding of the role of fibrosis in recovery for different HF etiologies.

Our study showed that heterogeneity of fibrosis is a valuable measure to predict the etiology of HF patients. We also evaluated many geometric features of fibrotic patches in the images of IC and NIC samples for their ability to predict etiology. Comparing these features revealed the compact grouping of NIC. The largest and mean area, as well as the length of the major and minor axis of the ellipse fitted to fibrotic patches were small in NIC. However, in IC these geometric features were widely spread, resulting in the inability to cluster all IC. This reflects not only the heterogeneity in HF but also the heterogeneity of fibrosis patterns, particularly in IC. Previously, a combination of different patterns of fibrosis was observed in a majority of NIC samples (Glashan et al., 2020). In contrast, our analysis of geometric features showed that IC has an even more pronounced variety of fibrosis patterns.

### Alteration of Propagation and Conduction Velocities Due to Fibrosis

In this study, we applied a novel characterization of CV vectors and their tilt from the expected direction of conduction. We found that though mean values of |CV| did not change between donor, IC, and NIC samples, their distribution differed. IC displayed the largest SD, followed by NIC, and donors displayed the smallest SD. The median |CV| decreased with increasing fibrosis and its heterogeneity (Table 3). Various studies on human hearts revealed the diversity of changes in the CV in different heart diseases, including fibrosis (Table 4). For example, CV decreased in both sub-endocardial and sub-epicardial LV in end-stage HF patients with NIC (Glukhov et al., 2012). In contrast, CV increased in DCM with fibrosis (Anderson et al., 1993). Though the primary factor for decreased CV in NIC was found to be the remodeling of connexin 43, the abnormal features in conduction, including discontinuities and CV alternans, were attributed to the interstitial fibrosis in HF vs. normal hearts.

**TABLE 4:** Clinical and experimental measures of CV in human cardiac ventricles.

**Normal**			**Disease**			**Disease type**	**Tissue type**	**References**
**L (cm/s)**	**T (cm/s)**	***N* (cm/s)**	**L (cm/s)**	**T (cm/s)**	***N* (cm/s)**			
56.1	14.6						LV epi	Doshi et al., 2015
			51.3 ± 4.6			Hypertrophy	Basal LV	Dhillon et al., 2013
		40 ± 2			28 ± 3	NIC end-stage HF	LV epi	Glukhov et al., 2012
		49 ± 2			39 ± 3	NIC end-stage HF	LV endo	Glukhov et al., 2012
					41–87	CM	RV free wall	Nanthakumar et al., 2007
60, 85						Post VT ablation	LV free wall	Yue et al., 2005
			58 ± 15	24 ± 4		Diffuse fibrosis (20.7 ± 13.7%)	LV/RV epi	Kawara et al., 2001
			57 ± 13	28 ± 7		Patchy fibrosis (21.8 ± 13.8%)	LV/RV epi	Kawara et al., 2001
			53 ± 19	39 ± 13		Stringy fibrosis (11.8 ± 2.7%)	LV/RV epi	Kawara et al., 2001
65	48	51	56	32	26	Ischemia	LV free wall	Taggart et al., 2000–2004
			66 ± 9	<20		DCM, congestive HF	LV/RV	Wu et al., 1998
			70	20		DCM	Papillary muscle	Bakker et al., 1996
80 ± 8	23 ± 3		84 ± 9	23 ± 3		DCM (fibrosis 4.6 ± 5.6%)	LV epi	Anderson et al., 1993
			90 ± 9			DCM (fibrosis 9.5 ± 7.8%)	LV epi	Anderson et al., 1993
			79	7		Infarction	Papillary muscle	Bakker et al., 1993
		19.86 ± 5.44			30.55 ± 7.58	Hypertrophy	Septum	Toyoshima et al., 1982
					45	Hypertrophy	LV free wall	Dam et al., 1972
		46.4 ± 2.7					LV free wall	Durrer et al., 1970
		43.4, 44.9					Septum	Durrer et al., 1970

*L: Longitudinal, T: Transverse, N: Transmural, CM: Cardiomyopathy, DCM: Dilated Cardiomyopathy, LV: Left ventricle, RV: Right ventricle, epi: sub-epicardial, endo: sub-endocardial.*

Conduction velocities in the ischemic LV free wall was found to decrease in all three directions vs. normal tissue (Taggart et al., 2000–2004). Increased transmural CV was observed in the ventricular septum in hypertrophic hearts, compared to decreased transmural CV in HF vs. normal hearts (Toyoshima et al., 1982). Conduction patterns and spatial heterogeneity of activation were correlated with fibrosis in DCM patients (Anderson et al., 1993). This study revealed three sub-groups of DCM patients with group 1 similar to the donor, group 2 with moderately disturbed conduction, and group 3 with severely distorted conduction patterns. The fibrosis in groups 1–3 were 4.6 ± 5.6, 9.5 ± 7.8, and 28.3 ± 21%. However, the specific patterns of fibrosis were not characterized. The only published data on associations between fibrosis patterns and CV suggested that CVL did not change with different amounts of fibrosis in NIC patients (Kawara et al., 2001). However, CVT decreased with higher fibrosis. Notably, anisotropy in stringy fibrosis was lowest, followed by patchy fibrosis and the highest anisotropy was for diffuse fibrosis (1.35 vs. 2.04 vs. 2.42, respectively).

Prior simulations of fibrotic human cardiac tissues generally showed a decrease in CV with increasing fibrosis. Several approaches were introduced to model the effects of fibrosis. Modeling diffuse fibrosis as non-excitable obstacles that randomly replaced myocytes in the computational mesh and by altering the myocyte model yielded a decrease in CV to 0.3 m/s at 40% fibrosis and 0.1 m/s at 65% fibrosis vs. normal CV of 0.7 m/s (Ten Tusscher and Panfilov, 2007). This approach was modified for stringy fibrosis by introducing unexcitable obstacles as parallel segments of varying lengths (Nezlobinsky et al., 2020). CVL was reduced more with shorter segments, while the CVT was more reduced with longer segments. Simulation with a myocyte model coupled with a fibroblast model and fibrosis determined by the fraction of random sites defined as fibroblasts showed decreasing CV with increasing fibrosis (Majumder et al., 2012). With stronger coupling between fibroblasts and myocytes, the decrease in CV occurred at lower fibrosis. In several simulations of IC samples, we observed high local CV in the vicinity of high local fibrosis levels. This local increase is explained by a source-sink mismatch. Regions with high fibrosis ahead in the direction of conduction reduce the intracellular sink considerably while the source is large.

We showed that ∠CV is strongly affected by *V*_*nm*_ and σ*_*intra,Vnm*_.* ∠CV provides a local measure of the wavefront curvature. With increasing fibrosis, |∠CV| increased and also |CV| decreased. CV decreases in a convex wavefront due to a larger sink, and at a concave wavefront CV increases due to a smaller sink compared to the source (Fast and Kléber, 2021). An increased curvature of the wavefront is reflected in more spread in |CV|. We showed that ∠CV increased from donors to NIC to IC corresponding to increasing SD in |CV|. Our results also indicate that curvature of the wavefront increased with increasing fibrosis, especially for longitudinal conduction. Local distortions in the wavefront arose due to underlying fibrotic patterns, which depended on the etiological origins of HF. Similar zig–zag patterns of conduction are well documented as precursors for reentry and slowed conduction fundamental to arrhythmogenesis (Bakker et al., 1993).

Linear regression analysis revealed that median |CVT| decreased (Figures 5G,K) and median |∠CVT| increased (Figures 5H,L) as *V*_*nm*_ and σ*_*intra,Vnm*_* increased (Table 3). However, visual inspection of the sample distribution showed that this relationship was caused by deviations from an otherwise constant distribution for *V*_*nm*_ > 40% and σ*_*intr*__*a*__,Vnm_* > 25%. The changes in median |CVT| and median |∠CVT| were negligible for *V_*nm*_* < 40% and σ*_intra,Vnm_*< 25%. The deviations were for IC samples, which stresses the importance of etiology-specific modeling.

We found that CV anisotropy was not affected by fibrosis. Median anisotropy was around ∼2 for donors and HF. The anisotropy is consistent with the observations in the normal myocardium (Spach et al., 1981). Prior measurements of CVL and CVT in various diseased LV in humans show anisotropy in the range of 1.5–3.4 (Table 4). A similar variation is observed in very limited measurements available from normal human hearts. Anisotropy was found to increase with increasing fibrosis at different rates depending on the dimensions of fibrotic patterns in the domain (Nezlobinsky et al., 2020). However, the combination of different patterns and dimensions of fibrosis within each patient introduces more complexity.

In our simulations, we observed that maximum upstroke velocity was lower during fast longitudinal conduction and higher during slow transverse conduction (Supplementary Figures 3A,B). The higher upstroke velocity along the transverse direction is due to less charge dissipation to sink. This is consistent with the current understanding of anisotropic conduction based on seminal experiments on normal canine cardiac tissue (Spach et al., 1981). The smaller sink provides a higher safety factor for transverse relative to longitudinal conduction providing a path for conduction to proceed even when longitudinal conduction is blocked (Valderrabano, 2007).

The pronounced etiology-dependent differences in |CV| and ∠CV indicate a need to adjust modeling parameters based on the clinical background of patients. Our study suggests that conduction fundamentally differs in two major etiologies of HF due to the characteristics of fibrosis. We showed that the etiology is reflected on the heterogeneity of fibrosis. We propose that including heterogeneity in the modeling of tissue of HF patients based on their clinical data is essential to uncover abnormalities of microscopic conduction. Our study stresses the clinical need to identify disease-specific fibrotic patterns in HF patients and stratify them based on the risk of arrhythmogenesis.

### Limitations

This study has several limitations. The tissue samples from patients undergoing LVAD implantation were procured close to the apex and had mixed orientation of myocytes. We minimized this effect by choosing a region of analysis from the image where the myocyte orientation is uniform. The samples were also distal from any scar and hence not confounded by scar fibrosis. We imaged a single slice for characterizing the fibrosis for each subject, which only coarsely represents the overall fibrosis and its heterogeneity in left ventricles. Analysis of larger areas would more comprehensively represent the heterogeneity in tissue remodeling as suggested in Wuelfers et al. (2021). The 2D imaging and modeling does not completely represent the transmural heterogeneity and anisotropy of the myocardium. Further sampling of ventricular tissue could reveal if the analysis of a small sample from any region can distinguish the etiology and also if the apical tissue is a good representative for the ventricular tissue. Due to limitations of tissue procurement from HF patients, a more extensive sampling was not feasible.

The bidomain electrophysiological simulations utilized the concept of homogenized media of myocyte and ES that coexist at every mesh element. However, a more microscopically realistic modeling approach could consider volumes of ES and myocytes as distinct spaces separated by a membrane (Roberts et al., 2008). Our detailed microscopic images form a basis for such modeling.

In this work, an explicit time-stepping scheme was utilized for simulations requiring a time resolution of 1e-8s for such a fine mesh grid. The computation time was ∼650 s for a mesh with 400 elements simulated for a duration of 15 ms. Using an implicit scheme might allow a coarser time resolution and shorter run time with similar accuracy. Due to the time required for simulations, we characterized conduction for a single wave through the domain. Investigations on the effect of basic cycle length on CV were not performed but might provide insights into arrhythmogenesis in heterogeneous domains. A second order polynomial model to interpret the relationship between CV and fibrosis values gives an increased *R*^2^. However, we do not have a good biophysical model describing the relationships between fibrosis measures and conduction. Hence, we applied a simple linear regression model to make coarse statements about the relationships. We lack functional measurements from this tissue to compare the results from simulations. However, we discussed our results in the context of published experimental data to arrive at conclusions.

## Materials and Methods

### Tissue Collection

The tissue collection and clinical characterization for this study were approved by the Institutional Review Board of the University of Utah Health, Intermountain Medical Center, Salt Lake City VA Medical Center, which are members of the Utah Transplantation Affiliated Hospitals Cardiac Transplant Program. Transmural tissue biopsies from the left ventricular apical region of HF with reduced ejection fraction (*n* = 16) and non-failing donor hearts (*n* = 5) were fixed immediately in 10% formalin. The biopsies were from the apical core of patients undergoing LVAD implantation or from matching locations in the failed heart during transplant and the donor hearts. The donor hearts were not suitable for transplantation due to non-cardiac reasons. The demographics and clinical data for donors and HF patients are summarized in Table 1.

### Tissue Processing

The fixed tissue samples were rinsed in phosphate buffered saline (PBS) within a day of fixation. The samples were then embedded in 3% agarose gel and sectioned using a vibratome Leica VT1200S (Leica Biosystems, Wetzlar, Germany) to obtain slices of 100 μm thickness. The midmyocardial slices were detached from agarose and washed in PBS before performing immunohistochemistry. We applied primary antibodies A5228 and V6630 (Sigma-Aldrich, St. Louis, MO, United States) at a concentration of 1:200 in a blocking solution with normal goat serum to bind to the proteins α-SMA and vimentin, respectively. Vimentin marked fibroblasts and endothelial cells in blood vessels. Smooth muscle cells, including myofibroblasts, were marked with α-SMA. Slices were incubated overnight on a rocker at room temperature. After washing with PBS three times, goat anti-mouse secondary antibodies A21137 and A21240 (Thermo Fisher Scientific, Waltham, MA, United States), conjugated to AF 555 and AF 647, respectively, were applied at 1:200 in the blocking solution to attach to the corresponding primary antibodies. We incubated these slices for 6 h at room temperature on the rocker together with 4′,6-diamidino-2-phenylindole (DAPI, D3571, Thermo Fisher Scientific) at 3 μg/ml to label the nuclei. After another set of three rinses of the slices in PBS, we applied WGA conjugated to a green fluorescent dye (CF488A, Biotium Inc., Fremont, CA, United States) at a concentration of 40 μg/mL in PBS to the slices for at least 4 h to label the glycocalyx and ECM proteins. Post incubation, the slices were washed with PBS and mounted on coverslips of 0.16–0.19 mm thickness with Fluoromount-G (#17984-25, Electron Microscopy Science, Hatfield, PA, United States) using a compression-free mounting method (Seidel et al., 2016). After curing for 24 h at a relative humidity between 30 and 35%, the samples were coated with a nail hardener before imaging. The humidity was controlled by placing the samples in a chamber with a bath of saturated NaI solution.

### Imaging and Image Processing

We imaged the coverslips with human cardiac tissue slices using a laser scanning confocal microscope Leica TCS SP8 (Leica Microsystems, Wetzlar, Germany) with a 40x oil immersion objective at a resolution of 378 nm per pixel. The imaging regions were chosen where the long-axis of myocytes were parallel to the X-axis of the coordinate axis. To cover areas of 1 mm^2^ and larger, several images of the size 1,024 × 1,024 pixels were stitched together using the merge tool in the post-processing software of the microscope (Leica Application Suite X 3.5.5). For this merging, imaging was performed with an overlap of 10% between adjacent images. The depth of imaging within the 100 μm slice was chosen to obtain uniform intensity throughout the area covered. We analyzed the signals in a region of interest of approximately 1 mm^2^ area chosen by avoiding edges of the section as well as blood vessels within the image (Figure 1A). The raw images of DAPI (Figure 1B), WGA (Figure 1C), vimentin (Figure 1D), and α-SMA (Figure 1E) were initially segmented using histogram-based thresholding as described in Seidel et al. (2016). WGA labeled collagen as well as extracellular membrane. To avoid the exclusion of regions from ES due to variation in intensities of collagen labeling, masks covering non-myocyte spaces were manually drawn and added to the thresholded WGA signal to obtain the segmented ES using Fiji (Schindelin et al., 2012). The α-SMA and vimentin signals were processed to avoid the labeling of endothelial and smooth muscle cells through a one-pixel opening followed by removing objects larger than 1,000 pixels from the thresholded images. The nuclei within one pixel of α-SMA or vimentin signal were incorporated as myofibroblasts or fibroblasts, respectively.

### Analysis of Fibrosis

We calculated *V*_*nm*_ as the fraction of the segmented non-myocyte space within the selected area in the image of each sample. We also calculated *V*_*f*_ and *V*_*mf*_ as the fraction occupied by fibroblasts and myofibroblasts, respectively. *V*_*e*_ was defined as the fraction of ES. The non-myocyte space was defined as the sum of *V_*e*_, V*_*f*,_ and *V_*mf*_.*


(1) $$V_{n\,m}=V_{e}+V_{f}+V_{m\,f}$$

Fibrosis in each HF sample was defined as the increase of the non-myocyte space from the average *V*_*nm*_ in donors.


(2) $$F\,i\,b\,r\,o\,s\,i\,s=V_{\mathrm{nm},H\,F}-m\,e\,a\,n\,\left(V_{n\,m,D\,o\,n\,o\,r\,s}\right)$$

We determined the group-wise *V*_*nm*_ from the images for donors and HF. The calculation of *V*_*nm*_ was repeated by dividing the image into 50 μm × 50 μm sub-images to find the intra-subject heterogeneity. The variability of intra-subject fibrosis σ*_*intra,Vnm*_* was calculated based on SD of *V*_*nm*_ of sub-sampled regions within each subject. We performed linear regression analyses of σ*_*intra,Vnm*_* with respect to *V*_*nm*_. We further separated HF into IC and NIC samples to assess *V*_*nm*_ and σ*_intra,Vnm._*

### Classification of Heart Failure and Association to Etiology

The HF samples were clustered based on different measures of fibrosis. The clusters were evaluated in their ability to differentiate their clinical etiology as IC and NIC. In addition to *V*_*nm*_ and σ*_*intra,Vnm*,_* features from images that capture the pattern and arrangement of fibrotic patches were used for classification. We processed the images to extract the connected components that are larger than 150 μm^2^ from the binary images of non-myocyte fractions. Among these objects, the object with the largest area and the mean area of objects were calculated. We calculated the eccentricity, major, and minor axes of an ellipse that has the same second central moment as the object for all the objects in each image. Eccentricity was defined as the ratio of the major to the minor axis. We compared these image features in IC vs. NIC, and features with significant differences were noted. Next, we performed binary clustering of subjects using the *k*-means algorithm with these image features, *V*_*nm*_ and σ*_*intra,Vnm*_* individually and in different combinations as the input. The algorithm was repeated 50 times for each clustering, and the clusters with the lowest sum of Euclidean distances of points to the cluster centroid were chosen as the result. To evaluate the clustering, the cluster with the lower centroid was assigned the label of NIC and that with the higher centroid was assigned IC. For clustering by σ*_*intra,Vnm*_* only, the mean of the two centroids was calculated as the value of σ*_*intra,Vnm*_* that can discriminate between IC and NIC. After assigning labels to the clusters, we calculated the positive and negative predictive value, accuracy, specificity, and sensitivity for the classification.

### Modeling of Fibrotic Tissue and Simulation

We utilized the bidomain model of cardiac tissue electrophysiology to characterize the electrical conduction in fibrotic tissue (Tung, 1978). In this model, the cardiac tissue is described with a myocyte and an extracellular domain. These two domains coexist at every point of space and are subject to principles of current conservation. Any current flowing out of one domain has to enter the second domain, and the net current is zero. Two Poisson equations describe the relationship between current and potential for each domain and the interaction between the two domains:


(3) $$\nabla \cdot \left(\sigma_{m\,y\,o}\,\nabla \phi_{m\,y\,o}\right)=-f_{s,m\,y\,o}+\beta_{m\,y\,o}\,I_{m\,y\,o,e}$$

(4) $$\nabla \cdot \left(\sigma_{e}\,\nabla \phi_{e}\right)=-f_{s,e}-\beta_{m\,y\,o}\,I_{m\,y\,o,e}$$

where σ_*myo*_ and σ_*e*_ are the electrical conductivity tensors (S/m) of the myocyte and extracellular domain, respectively, and ϕ*_*myo*_* and ϕ*_*e*_* are the electrical potentials (V) of the myocyte and extracellular domain, respectively. *f*_*s,myo*,_ and *f*_*s,e*_ are the current source densities (A/m^3^) for the myocyte and extracellular domain, respectively. Current flowing between the myocyte and extracellular domain, *I*_*myo,e*_ (A) was calculated using the electrophysiological model of a normal human ventricular myocyte (Tusscher et al., 2004).


(5) $$I_{m\,y\,o,e}=I_{i\,o\,n}+C_{m}\,\frac{\partial V_{m,m\,y\,o}}{\partial t}$$

where *I*_*ion*_ (A) is the total ion channel current flowing through the membrane of a myocyte and *C_m_* = 2e7nF is the membrane capacitance per myocyte. Membrane voltage of myocytes *V*_*m,myo*_ (V) was defined as the difference between ϕ*_*myo*_* and ϕ*_*e*_*. *I*_*ion*_ defined by the biophysical model of human ventricular myocyte includes fast Na^+^ current (*I*_*Na*_), L-type Ca^2+^ current (*I*_*CaL*_), transient outward current (*I*_*to*_), rapid delayed rectifier current (*I*_*Kr*_), slow delayed rectifier current (*I*_*Ks*_), inward rectifier K current (*I*_*K1*_), Na^+^/Ca^2+^ exchanger current (*I*_*NaCa*_), Na^+^/K^+^ pump current (*I*_*NaK*_), plateau Ca^2+^ and K^+^ currents (*I*_*pK*_ and *I*_*pCa*_), and background Ca^2+^ and K^+^ currents (*I*_*bCa*_ and *I*_*bK*_). These individual membrane currents are defined based on the specific channel conductances and transmembrane voltage.


(6) $$I_{i\,o\,n}=I_{N\,a}+I_{K\,1}+I_{t\,o}+I_{K\,r}+I_{K\,s}+I_{C\,a\,L}+I_{N\,a\,C\,a}+I_{N\,a\,K}+I_{p\,C\,a}+I_{p\,K}+I_{b\,C\,a}+I_{b\,N\,a}$$

The conductivity tensors for myocyte and extracellular domains were defined by a linear relationship with their respective volume fractions, *V*_*myo*_ and *V*_*e*_ (dimensionless):


(7) $$\sigma_{m\,y\,o}=V_{m\,y\,o}\,{\bar{\sigma }}_{m\,y\,o}$$

(8) $$\sigma_{e}=V_{e}\,{\bar{\sigma }}_{e}\,$$

where the tensors σ*_*myo*_* = 0.5 S/m and σ*_*e*_* = 1 S/m described longitudinal conductivity for the volume fraction of 100% for the respective domains. The longitudinal to transverse anisotropy of the conductivity with respect to the myocyte orientation was 10 and 2 for intra- and extracellular domains, respectively. We defined the relationship between the volume fractions of bidomain domains as:


(9) $$V_{m\,y\,o}=1-V_{e}-V_{f}-V_{m\,f}$$

where *V*_*e*_, *V*_*f*,_ and *V*_*mf*_ are the volumes equivalent to the fractional areas of ES, fibroblasts, and myofibroblasts, respectively, calculated for each mesh element. Since the total area occupied by fibroblasts and myofibroblasts combined was within 5% and intercellular coupling through gap junctions was neglected, they were not considered to form a separate domain. Their fractional space in the images translated to a non-conductive fraction in the mesh. Hence the conductivities associated with *V*_*f*_ and *V*_*mf*_ are zero. The number of myocytes per unit volume β*_*myo*_* (1/m^3^) was defined as:


(10) $$\beta_{m\,y\,o}=\frac{V_{m\,y\,o}}{V_{m\,y\,o,s\,i\,n\,g\,l\,e}}$$

where *V*_*myo,single*_ is the individual volume for a myocyte. *V*_*myo,single*_ = 41,073 μm^3^ was set to reflect the volume of a human myocyte (Gerdes et al., 1992).

2D meshes for bidomain cardiac electrophysiological simulation of fibrotic tissue were generated by selecting rectangular regions from the segmented binary WGA labeling of tissue images. The volume-based quantities in the domain are defined for 2D by treating the third dimension as having a unit length. The variation of fibrosis calculated for each image was incorporated by varying *V*_*e*_, *V*_*f*_, and *V*_*mf*_, at each element of the mesh according to the underlying image intensities. We downsampled each image such that the area covered by each mesh element is 2,500 μm^2^ of the image. The simulations were run for 15 ms with a time step resolution of 1e-8s. The rectangular domains of around 1 mm^2^ with a regular grid and an element edge length of 50 μm were activated using Dirichlet boundary conditions that held the transmembrane voltage of one edge of the domain at 1 mV for 2 ms after allowing the system to equilibrate for 2 ms. The extracellular and intracellular source currents were zero. The domain was subjected to no flux Neumann boundary condition.

We applied an implementation of the bidomain model (Seemann et al., 2010) that uses the Portable, Extensible Toolkit for Scientific Computation (Balay et al., 1997) to solve the Poisson equations. The Eqs 3, 4 are recast as an elliptic PDE (Eq. 11) and a parabolic PDE (Eq. 12):


(11) $$\nabla \cdot \left(\left(\sigma_{m\,y\,o}+\sigma_{e}\right)\,\nabla \Phi_{e}\right)=-\nabla \cdot \left(\sigma_{m\,y\,o}\,\nabla V_{m,m\,y\,o}\right)$$

$$\nabla \cdot \left(\sigma_{m\,y\,o}\,\nabla V_{m,m\,y\,o}\right)+\nabla \cdot \left(\sigma_{m\,y\,o}\,\nabla \Phi_{e}\right)$$

(12) $$\;\;=\beta_{m\,y\,o}\left(C_{m}\frac{\text{d}\,V_{m,m\,y\,o}}{\text{d}\,t}+I_{i\,o\,n}\right)$$

This implementation utilizes an operator splitting method with a forward Euler scheme for time-stepping. This modeling framework was benchmarked against other frameworks through monodomain simulations [acCELLerate indexed I in Niederer et al. (2011)]. We performed the calculations at each time step with the Generalized Minimal Residual method and additive Schwarz preconditioner to solve the linear system of equations limiting the relative and absolute tolerance to 1e-12.

The orientation of the myocytes combined with the placement of stimulus was used to determine the direction of conduction as longitudinal or transverse. All the images in which the long axes of myocytes were visualized were simulated with the activation on all four edges of the domain (*n* = 16). These images were rotated to align the long axis of myocytes along the X-axis. The two simulations with stimulus normal to the long axis were used to evaluate the longitudinal conduction. The other two simulations where the activation edge was parallel to the long axis were analyzed as transverse conduction. Only transverse conduction was evaluated when the image captured the axial cross-section of myocytes (*n* = 5).

### Evaluation of Conduction

The activation times at every element of the domains were determined to identify cases with regions of conduction block. The activation time was defined as the time of maximum upstroke velocity of the membrane potential within the range of -60 and 0 mV. We recorded the maximum upstroke velocities at four sets of three adjacent grid points spread uniformly over the mesh. Each set of three grid points are placed so that two of them lie along the X-axis and two of them lie along the Y-axis, with one point common to both pairs. The activation times at these points were also used to calculate the CV as described below applicable for regular rectangular grids. The magnitude and direction of the CV vectors, CVL and CVT, were measured for transverse and longitudinal conduction, respectively, according to


(13) $$\left|C\,V\right|=\frac{l}{\sqrt{t\,x^{2}+t\,y^{2}}}$$

and


(14) ∠⁢C⁢V={+x:tan-1⁡t⁢yt⁢x-x:180-∘tan-1t⁢yt⁢x+y:90-∘tan-1t⁢yt⁢x-y:90+∘tan-1t⁢yt⁢x

where *tx* and *ty* are the activation time differences between the two adjacent grid points along the X- and Y-axis, respectively. The distance between two adjacent grid points, *l*, was 50 μm. *x* and *y* correspond to longitudinal and transverse conduction. + corresponds to the left to right or top to bottom while *–* corresponds to the conduction in opposite directions. The simulations in two opposing directions of longitudinal and transverse conduction were separately analyzed to quantify the effect of direction in the image-based mesh. They were pooled together to analyze the conduction with respect to different groups and the relationship between the conduction and fibrosis. |∠CV| was calculated to measure the tilt of CV vectors.

To evaluate the effect of the boundary on CV, we performed the simulation of a donor sample mesh embedded in a homogenous mesh (Supplementary Figure 4). We calculated the space constants of the domain, λ_*l*_ and λ_*t*_ for the longitudinal and transverse directions, respectively, according to Roth (1997).


(15) $$\lambda_{l}=\sqrt{\frac{R}{\beta }\,\frac{\left(\sigma_{m\,y\,o\,l}\,\sigma_{e\,l}\right)}{\left(\sigma_{m\,y\,o\,l}+\sigma_{e\,l}\right)}}$$

(16) $$\lambda_{t}=\sqrt{\frac{R}{\beta }\,\frac{\left(\sigma_{m\,y\,o\,t}\,\sigma_{e\,t}\right)}{\left(\sigma_{m\,y\,o\,t}+\sigma_{e\,t}\right)}}$$

where *R* = 0.2 Ωm^2^ is the membrane resistance and β = 2e5 m^–1^ is the myocyte surface-to-volume ratio (Tusscher et al., 2004). In our simulations, we have set the conductivities for 100% volume fractions, σ*_*myol*_* = 0.1 S/m, σ*_*el*_* = 1 S/m, σ*_*myot*_* = 0.01 S/m, and σ*_*et*_* = 0.5 S/m. Thus, λ*_*l*_* = 0.30 mm and λ*_*t*_* = 0.09 mm.

Each element of the homogenous mesh was composed of volume fractions that are the mean value of the volume fractions of elements in the donor mesh. The dimensions of this domain were 5 mm in X direction and 1 mm in Y direction, with the donor mesh (Supplementary Figure 4A) forming the middle part of the new domain from 2 to 3 mm (Supplementary Figure 4B). |CV| and ∠CV calculated from longitudinal propagation with voltage clamping at the left end or right end of the domain were compared with those from the original donor mesh. The evaluation of CV at the same positions as the original mesh was now >2 mm from the boundary which is more than five times λ*_*l*_*. We evaluated mean error and root mean square error percent to determine the effect.

### Statistical Analysis

All statistical analyses were performed in MATLAB 2020a or later (Mathworks, Natick, MA, United States). Student’s *t*-test for unequal variances was performed to compare the composition of fibrosis in donors and HF, and *p* < 0.05 was considered significant. The ratio of variance in HF to that in donor for all the variables considered for *t*-test with unequal variances was larger than 5. Violin plots were generated using external code developed for MATLAB (Jonas, 2008). Inter-subject and intra-subject heterogeneity in fibrosis between IC, NIC, and donors were compared using one-way ANOVA, and *p* < 0.05 was considered significant. Boxes in the boxplots represented interquartile range, and whiskers extend to median ± 2.7 SD. Values beyond whiskers were considered outliers. All values for fibrosis characterization and CV were reported as mean ± SD. The variances between magnitude and tilt angles of CV were compared between different groups using a two-sample *f*-test. Regression analysis was performed using a linear model of the form *y* = *Ax* + *B*. The model was compared to a constant model and evaluated using the coefficient of determination (*R*^2^). *p* < 0.05 was considered significant.

## Data Availability Statement

The datasets presented in this study can be found in online repositories. The names of the repository/repositories and accession number(s) can be found below: Hive, University of Utah, https://doi.org/10.7278/S50D-BPS8-R06S.

## Ethics Statement

The studies involving human participants were reviewed and approved by IRB, University of Utah. The patients/participants provided their written informed consent to participate in this study.

## Author Contributions

ACS: sample preparation, imaging, analyses, manuscript draft, and revision. JG and JB: analyses and manuscript revision. JRV: sample acquisition, preparation, and manuscript revision. TSS: sample acquisition and preparation. CPK: sample, acquisition, preparation, and clinical data preparation. SGD: donor identification and manuscript revision. FBS: study design, manuscript draft, and revision. All authors contributed to the article and approved the submitted version.

## Conflict of Interest

The authors declare that the research was conducted in the absence of any commercial or financial relationships that could be construed as a potential conflict of interest.

## Publisher’s Note

All claims expressed in this article are solely those of the authors and do not necessarily represent those of their affiliated organizations, or those of the publisher, the editors and the reviewers. Any product that may be evaluated in this article, or claim that may be made by its manufacturer, is not guaranteed or endorsed by the publisher.
