# Supplementary material for: Etiology-Specific Remodeling in Ventricular Tissue of Heart Failure Patients and Its Implications for Computational Modeling of Electrical Conduction
Source: Front Physiol. 2021 Oct 5;12:730933. doi: 10.3389/fphys.2021.730933 (PMC8523803; doi:10.3389/fphys.2021.730933)
Supplement: Supplementary file 1 [file Data_Sheet_1.pdf]

Supplementary Material

1 Supplementary Figures

S1 Fig. Fractional space of fibrotic components. Fractional space occupied by (A) myofibroblasts and (B) fibroblasts. Differences were not significant between donor and HF.

S2 Fig. Comparison of CV vectors with conduction in opposite directions. (A) |CVL| in blue corresponding to conduction from left to right and that in green corresponding to conduction from right to left is mostly symmetric for donors and not symmetric for NIC. This symmetry for donors is also reflected in (B) *L*CVL. (C) |CVT| for conduction from top to bottom in blue and bottom to top in green has similar symmetry in donors and a lack of it NIC (D). The differences in *L*CVT between the two opposite directions are pronounced in IC.

S3 Fig. Probability distribution of maximum upstroke velocity in (A) longitudinal and (B) transverse conduction revealed a peak in the donors at ~200V/s and ~250V/s, respectively. The distributions displayed large spreads in IC, lesser variation in NIC, and very minimal spread in donors. (C-D) $\sigma_{|CVL|}$ and $\sigma_{|CVT|}$ did not change with increasing $\sigma_{intra\text{-}subject,Vnon\text{-}myo}$. (E-F) $\sigma_{|LCVL|}$ and $\sigma_{|LCVT|}$ have strong relationships with $\sigma_{intra\text{-}subject,Vnon\text{-}myo}$. (G-H) Median CV anisotropy was always between 1.5 to 3 and did not change with the non-myocyte fraction or its heterogeneity. Legend in (C) applies to (D-H).

S4 Fig. Boundary effects in CV assessment. (A) Original mesh of a donor tissue sample used in analysis and its CV vectors. (B) A homogenous domain of 5mm in x direction and 1mm in y direction , with the donor mesh in (A) forming the middle part of the new domain in from 2-3mm and the CV vectors in longitudinal directions at same locations as in (A).

Supplementary Movies

Supplementary Movie 1. Simulation of longitudinal conduction in donor sample. The corresponding contour plot of activation times is presented in Fig 4A.

Supplementary Movie 2. Simulation of longitudinal conduction in NIC sample. The corresponding contour plot of activation times is presented in Fig 4D.

Supplementary Movie 3. Simulation of longitudinal conduction in IC sample. The corresponding contour plot of activation times is presented in Fig 4G.
