# Supplementary figures and images for: Etiology-Specific Remodeling in Ventricular Tissue of Heart Failure Patients and Its Implications for Computational Modeling of Electrical Conduction
Source: Front Physiol. 2021 Oct 5;12:730933. doi: 10.3389/fphys.2021.730933 (PMC8523803; doi:10.3389/fphys.2021.730933)

Fig. S1

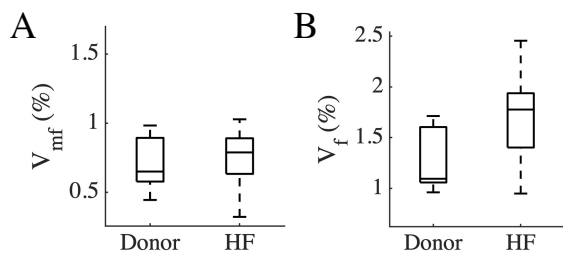

Fig. S2

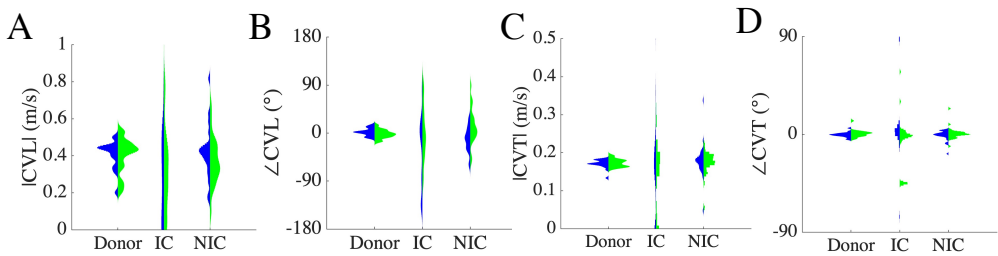

Fig. S3

A

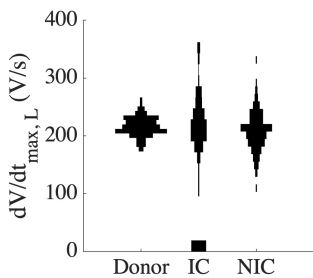

B

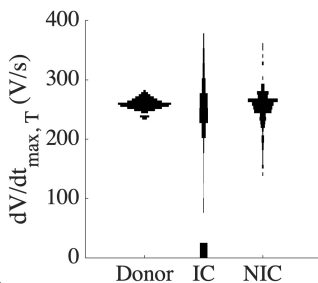

C

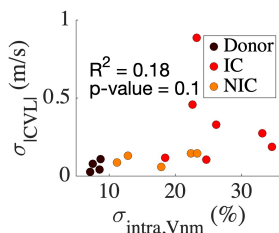

D

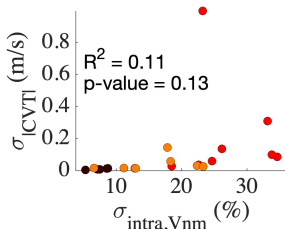

E

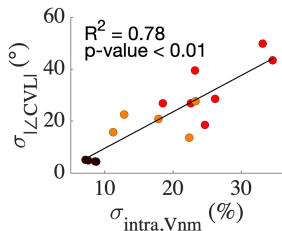

F

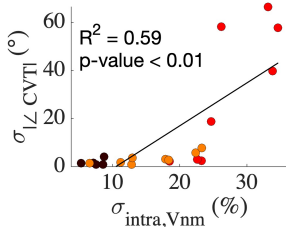

G

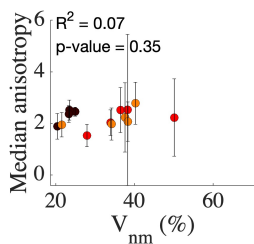

H

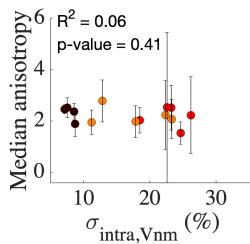

Fig. S4

A

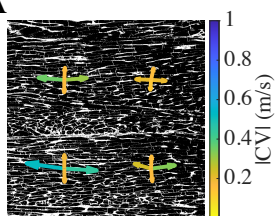

B

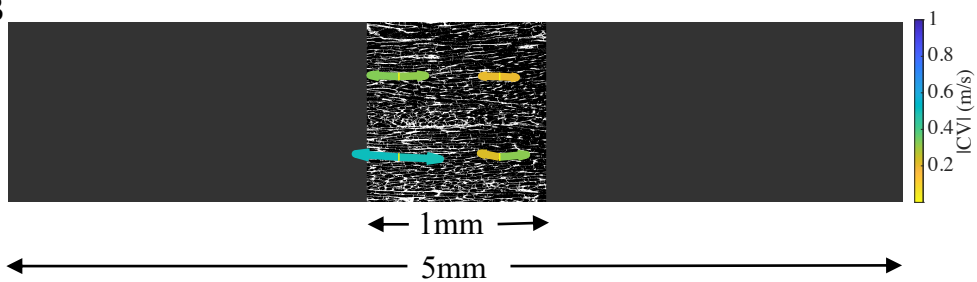

Supplement: Supplementary file 2 [file Image_1.PDF]
